# A case report of cardiogenic shock with transcranial color-coded ultrasound monitoring under ECMO

**DOI:** 10.3389/fcvm.2026.1844563

**Published:** 2026-09-03

**Authors:** Yuyi Chen, Jingyi Wu, Huiying Chen, Jiefeng Xie, Yuping Liao, Qianwei Zheng, Yu Gan

**Affiliations:** 1 Health Management Center, The Tenth Affiliated Hospital of Southern Medical University (Dongguan People’s Hospital), Dongguan, Guangdong, China; 2 Guangdong Medical University, Zhanjiang, Guangdong, China; 3 Department of Critical Care Medicine, The Tenth Affiliated Hospital of Southern Medical University (Dongguan People’s Hospital), Dongguan, Guangdong, China

**Keywords:** cardiogenic shock, case report, cerebral perfusion monitoring, neuroprotection, transcranial color-coded sonography, VA-ECMO

## Abstract

**Background:**

Cardiogenic shock (CS) during venoarterial extracorporeal membrane oxygenation (VA-ECMO) support carries high mortality, often due to neurological injury. Precise matching of ECMO flow to cerebral perfusion is critical. Transcranial color-coded sonography (TCCS) offers real-time, non-invasive hemodynamic assessment to guide flow titration, though its clinical integration remains limited.

**Case presentation:**

This study reports a 58-year-old male firefighter who suffered out-of-hospital cardiac arrest due to acute myocardial infarction, resulting in CS, right frontal lobe hemorrhage, and hypoxic-ischemic encephalopathy. VA-ECMO was initiated on-site and maintained post-resuscitation alongside intra-aortic balloon pump (IABP) and continuous renal replacement therapy (CRRT). TCCS was used throughout hospitalization to monitor middle cerebral artery flow parameters [including pulsatility index (PI), resistivity index (RI), and non-invasive intracranial pressure (nICP)]. These flow indexes were used to guide dynamic ECMO flow adjustments (gradually decreased from 3.25 L/min to 2.12 L/min) to preserve cerebral perfusion within target ranges. At the three-month follow-up, his neurological function recovered, and he was living independently. Six-month follow-up revealed progressive resolution of the intracranial hemorrhage.

**Conclusion:**

This case demonstrates the feasibility of TCCS-guided neuromonitoring during ECMO support for CS. TCCS provided real-time cerebral hemodynamic data that informed bedside ECMO flow titration. Whether this approach mitigates secondary brain injury or improves neurological outcomes requires further validation.

## Introduction

Cardiogenic shock (CS) is a life-threatening condition resulting from severe cardiac pump failure, leading to inadequate tissue perfusion, multi-organ dysfunction, and high mortality. Acute myocardial infarction (AMI) is the most common cause (1), and diagnosis requires persistent hypotension, low cardiac index, elevated filling pressures, and signs of hypoperfusion (2). The cornerstone of CS management is hemodynamic stabilization and treatment of the underlying cause (3). Despite optimal medical therapy, 10%–15% of patients develop refractory shock, necessitating mechanical circulatory support (MCS) (4). Extracorporeal membrane oxygenation (ECMO), as an effective equipment, provides critical hemodynamic support and is recommended in advanced CS (5). However, neurological injury occurs in up to 78% of ECMO patients (median 13%) and is a leading cause of death, with mortality rising to 83% when present (6, 7). This risk is heightened in venoarterial (VA)-ECMO due to non-pulsatile flow, which impairs cerebral autoregulation and predisposes to ischemic or hemorrhagic brain injury (8, 9). Therefore, precisely matching ECMO flow to cerebral perfusion demands is paramount for reducing complications and improving survival (10).

TCCS enables real-time, non-invasive assessment of cerebral hemodynamics, including flow velocities, pulsatility index (PI), and estimated intracranial pressure (ICP) (11). In TCCS, the PI reflects downstream cerebrovascular resistance, an elevated PI often suggests increased intracranial pressure or impaired compliance, while a very low PI may indicate loss of arterial pulsatility. Non-invasive ICP estimates are frequently derived from PI using validated formulas. It has shown promise in guiding ECMO flow titration to match cerebral perfusion demands, potentially mitigating neurological complications (12). Yet, clinical integration of TCCS for neuromonitoring during ECMO remains limited, especially in adult CS populations. Evidence supporting its utility in individualizing hemodynamic management and improving neurologic outcomes is still emerging (13). Therefore, further case-based observations are needed to clarify the role of TCCS in optimizing cerebral perfusion and enhancing survival in high-risk ECMO-supported CS patients. This report presents such a case, illustrating the feasibility of TCCS-guided ECMO management and its potential to inform individualized hemodynamic support.

## Case description

### Initial presentation and clinical course

A 58-year-old male firefighter was found unconscious with convulsions at approximately 09:00 on August 15, 2022 without identifiable precipitating factors. On-site resuscitation included automated external defibrillator (AED) use and chest compressions. Paramedics diagnosed cardiorespiratory arrest and initiated advanced life support, including mechanical chest compressions, endotracheal intubation with ventilatory support, repeated defibrillation, intravenous epinephrine, and alkalinization therapy. However, return of spontaneous circulation was not achieved. The medical team of the Tenth Affiliated Hospital of Southern Medical University arrived on site at 10:20 AM, and the patient was transferred to our department after successful ECMO initiation at 10:45 AM. The 12-lead electrocardiogram (ECG) revealed ST-segment elevation in the inferior leads, consistent with an acute ST-segment elevation myocardial infarction (STEMI) **(**Supplementary Figure S1**)**.

**Figure 1:**
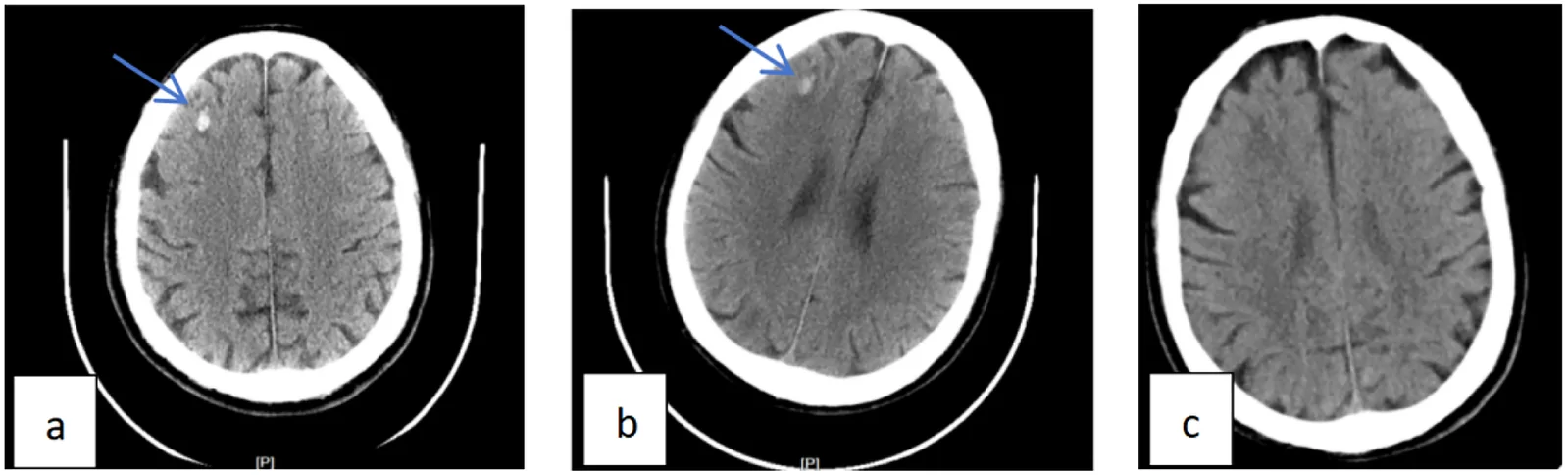
Serial cranial CT scans of the patient. **(a)** August 15: intracerebral hemorrhage in the right frontal lobe (blue arrows indicate the site of intracerebral hemorrhage). **(b)** August 22 (7-day follow-up): partial resolution of the hemorrhage (blue arrows indicate the site of intracerebral hemorrhage). **(c)** September 2 (18-day follow-up): near-complete absorption of the hemorrhage.

**Figure 2:**
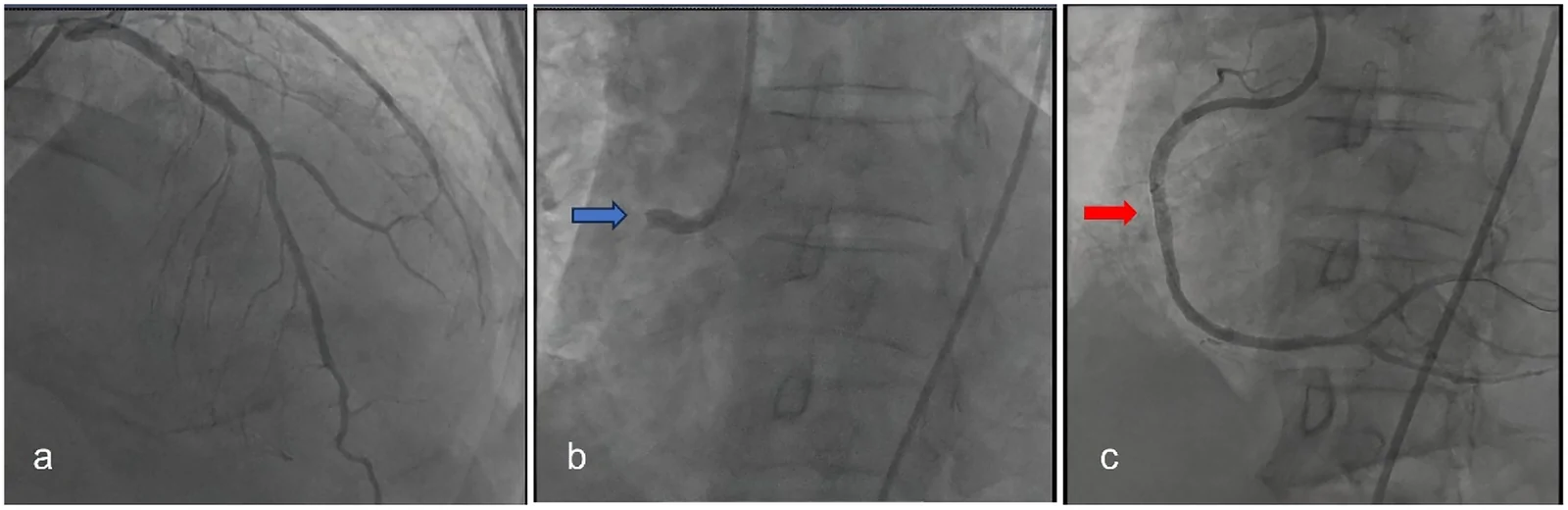
Preoperative and postoperative coronary angiography results. **(a)** Good condition of the left main and anterior descending artery. **(b)** Complete occlusion of the right coronary artery. **(c)** Improved arterial blood flow after balloon dilation and stent placement in the right coronary artery.

The patient was afebrile (37.0 °C) with a pulse rate of 100 beats/min and a ventilator-assisted respiratory rate of 15 breaths/min; blood pressure was unmeasurable. The patient was comatose with a Glasgow Coma Scale score of 3 and was mechanically ventilated via an endotracheal tube, appearing pale. Upon admission, diagnostic evaluation included coronary angiography, which revealed coronary artery occlusion, and a non-contrast head computed tomography (CT) demonstrated hemorrhage in the right frontal lobe (Figure 1a). The preliminary diagnoses were: (1) Cardiorespiratory arrest; (2) Suspected acute myocardial infarction; (3) Post-cardiopulmonary resuscitation state; (4) Right frontal lobe hemorrhage; and (5) Hypoxic-ischemic encephalopathy. At the same time, Continuous Renal Replacement Therapy (CRRT) was immediately initiated to correct acidosis and hypokalemia. Ventricular fibrillation occurred at 1:00 PM and was successfully treated with electrical cardioversion, resulting in a sinus rhythm of 134 beats/min and a blood pressure of 74/40 mmHg (maintained with norepinephrine and dopamine). Coronary intervention was performed at 5:00 PM. The left main coronary artery (LCA), left anterior descending (LAD), and left circumflex artery showed good blood flow, but the right coronary artery (RCA) was completely occluded (Figures 2a,b), during the coronary angiography and stent implantation procedure, intra-aortic balloon pump (IABP) was inserted at 18:15 on August 15, and 3,000 units of heparin was administered, and no further systemic anticoagulation was used thereafter. After RCA revascularization, blood flow was successfully restored after balloon angioplasty and stent implantation (Figure 2c).

**Figure 3:**
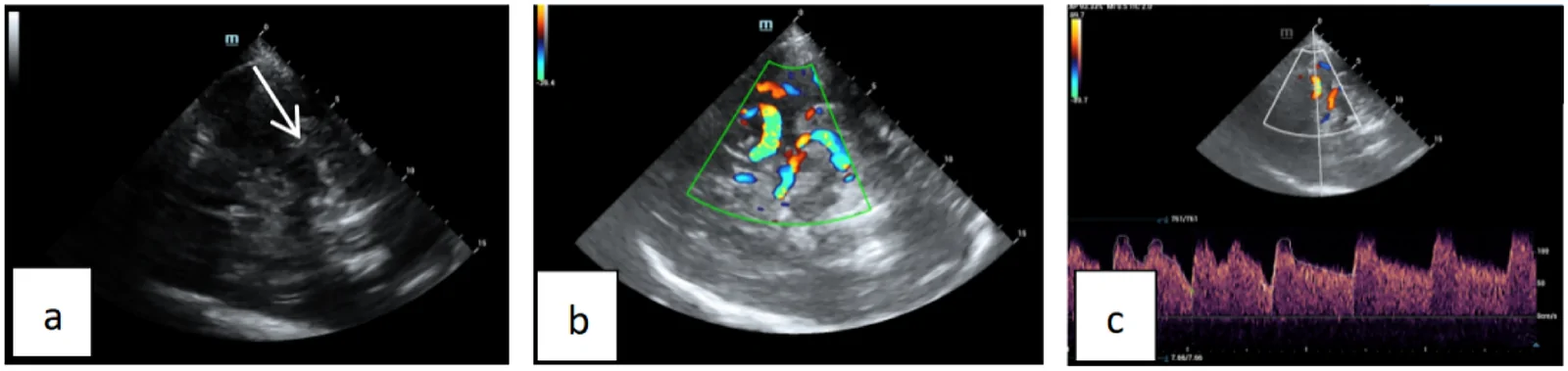
Transcranial color-coded sonography (TCCS) monitoring procedure. **(a)** Identification of the midbrain plane, the most basal anatomical level, showing the characteristic butterfly-shaped midbrain. The contralateral skull (typically 12–15 cm away) was used to confirm an adequate ultrasound window. **(b)** The middle cerebral artery selected as the target vessel for monitoring. **(c)** TCCS-based monitoring of cerebral hemodynamic parameters and estimation of noninvasive intracranial pressure (nICP) to guide ECMO flow adjustment. **(d)** ECMO flow rate change during treatment period. RI: resistive index; PI: palsatility index; RMCA: right middle cerebral artery; LMCA: left middle cerebral artery; ECMO: extracorporeal membrane oxygenation.

**Figure 4:**
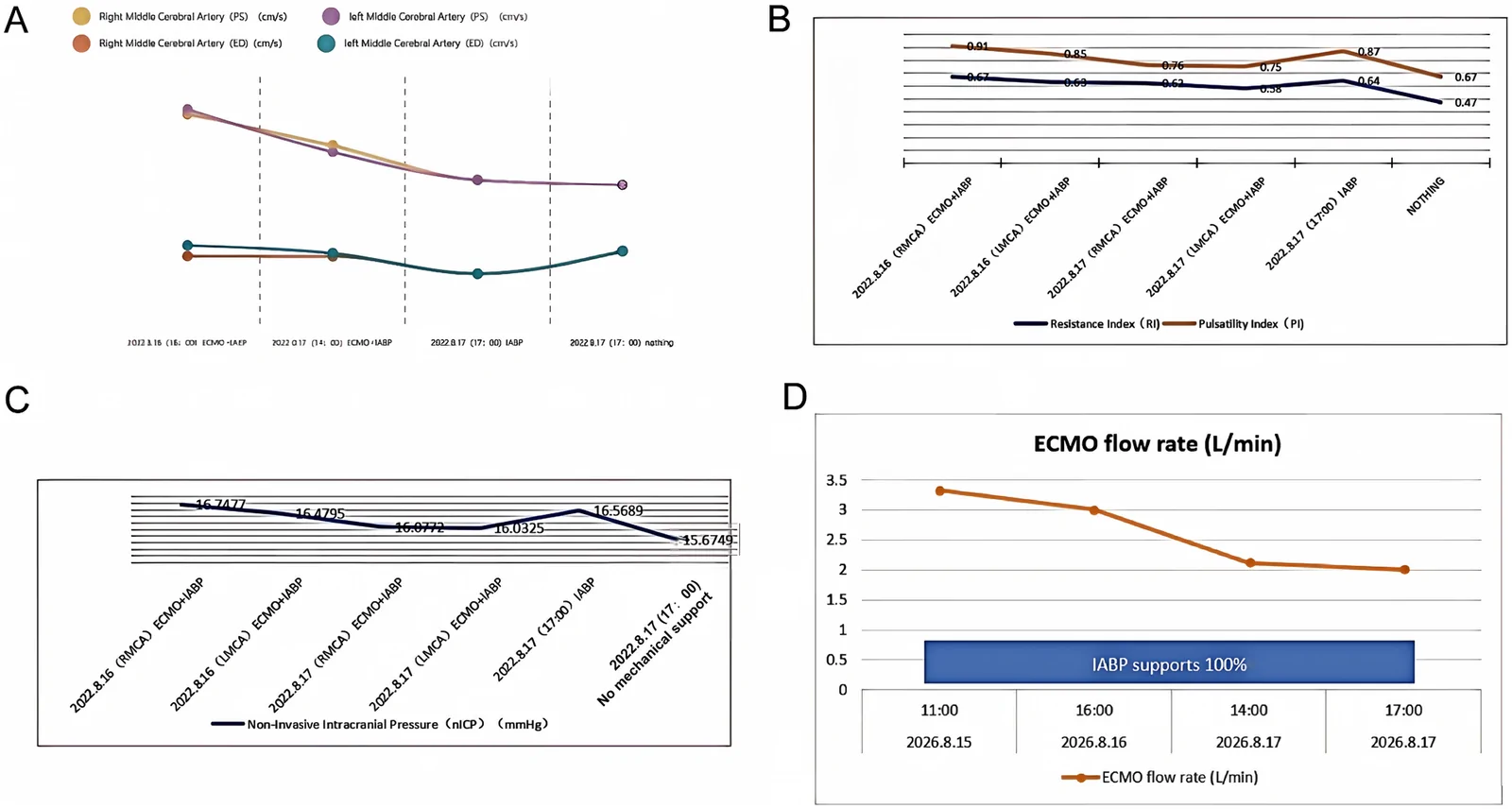
TCCS monitoring of cerebral hemodynamic parameters during ECMO and IABP support. **(a)** Systolic and diastolic blood flow velocities in the bilateral middle cerebral arteries remained symmetrical and stable. ECMO flow and IABP support were adjusted with improving cardiac function and blood pressure to avoid cerebral hypo- or hyperperfusion. After withdrawal of ECMO and IABP on August 17, cerebral perfusion showed minimal fluctuation. **(b)** Pulsatility index (PI) and resistance index (RI) remained stable during ECMO and IABP support but decreased after IABP withdrawal. **(c)** Noninvasive intracranial pressure (nICP) remained stable during ECMO and IABP adjustment. After cardiac recovery and ECMO withdrawal, nICP under IABP alone was higher than that without IABP support. IABP: intra-aortic balloon pump.

After coronary intervention, the patient still maintained ECMO, IABP, and CRRT. Subsequently, the patient's urine output improved, blood pressure gradually increased, and the lactate level progressively resolved from >15 mmol/L at 12:00 on August 15 to 1.4 mmol/L by 14:00 on August 16. On the afternoon of August 16th at 14:00, his blood pressure rose to 124/70 mmHg with a heart rate of 84 beats per minute. ECMO was successfully removed the following day, on the afternoon of August 17th at 15:00. CRRT was discontinued at 7:00 PM on August 16th, and ECMO was stopped at 3:00 PM on August 17th. The decision to wean from ECMO was based on the following criteria: (1) sustained hemodynamic stability without vasopressor escalation; (2) normalized serum lactate (<1.5 mmol/L) indicating adequate end-organ perfusion; (3) echocardiographic demonstration of recovered left ventricular systolic function with a left ventricular ejection fraction of 52% after IABP cessation; and (4) successful tolerance of reduced ECMO flow rates (1.5–2.0 L/min) for over 24 h without evidence of hypotension, recurrent lactate elevation, or signs of tissue hypoperfusion. Throughout the whole treatment process, regular TCCS monitoring was performed to dynamically adjust ECMO flow, maintaining stable intracranial perfusion and normal ICP. TCCS was performed using a Mindray Resona 7s system with the SPS-1U probe (1.6–2.0 MHz) via the bilateral temporal windows. Bilateral middle cerebral artery (MCA) was selected as the target vessel (Figure 3). During monitoring, systolic and diastolic velocities, mean flow velocity, PI, Resistivity Index (RI), non-invasive ICP (nICP), and ECMO flow rate were also recorded. The images were acquired at a depth of 50–60 mm (in 2-mm increments) with a sample volume of 10 mm via the temporal window. Angle correction (≤30°) was applied under TCCS color-flow guidance. All measurements were obtained from an average of at least five consecutive stable cardiac cycles by a single trained operator and were subsequently verified by a second experienced sonographer (13–16). nICP was calculated using the Czosnyka model (17): nICP = PI × 4.47 + 12.68. To guide ECMO therapy, target ranges were set: PI 0.65–1.1, nICP 15.59–17.60 mmHg, with adjustments made if PI < 0.5 or nICP > 20 mmHg. For this patient, ECMO flow was gradually decreased from 3.25 L/min to 2.12 L/min over ∼24 h, maintaining PI, RI, and nICP within target ranges (Figure 4). During and after ECMO support, cerebral perfusion remained stable, with slightly higher ICP observed under IABP alone. TCCS findings were interpreted together with systemic and respiratory variables, including arterial blood pressure, PaCO₂, PaO₂, hemoglobin, lactate, vasoactive support, ECMO flow, and IABP status. ECMO flow was adjusted cautiously according to the overall cerebral perfusion pattern rather than on the basis of a single PI or estimated nICP threshold. The individualized bedside decision process is summarized in Supplementary Figure S2.

During monitoring, systolic and diastolic flow velocities in the bilateral common carotid arteries were well-matched. The PI, RI, nICP remained stable (Figure 4; Table 1; Supplementary Table S1). Cerebral perfusion showed minimal fluctuation after ECMO decannulation. ICP was slightly higher with IABP support alone compared to the unsupported state.

**Table 1:** Detailed information regarding hemodynamic parameters at different time points.

Date	Time	PI	RI	nICP (mmHg)	MAP (mmHg)	Dopamine (mL)	Norepinephrine (mL)	ECMO Flow (L/min)	Support Mode		IABP	PaCO2(mmol/L)	PaO2(mmol/L)
2022/8/16	16:00	0.91	0.87	16.75	74	5	1	3.01	ECMO + IABP		100%	40	200
2022/8/17	14:00	0.85	0.76	16.48	101	5	0.6	2.12	ECMO + IABP (weaning)		100%	39	140
2022/8/17	17:00	0.75	0.67	16.03	86	5	0.4	/	IABP only		100%	41	139
2022/8/17	17:00	0.67	0.64	15.67	85	5	0.4	/	No mechanical support			37	160

Follow-up cranial CT scans demonstrated progressive resolution of the right frontal lobe hemorrhage at 7 days (August 22; Figure 1B) and near-complete resolution by September 2 (Figure 1C). The patient was discharged on August 25 with full independence in activities of daily living. At the three-month follow-up, he maintained complete functional independence without neurological sequelae.

## Discussion

This study report a 58-year-old man with cardiogenic shock post-cardiac arrest due to acute myocardial infarction, requiring VA-ECMO. He developed a right frontal lobe hemorrhage and hypoxic-ischemic encephalopathy. TCCS-guided ECMO flow titration maintained stable cerebral perfusion and normal intracranial pressure. He recovered fully and was discharged independently at three months. This case demonstrates TCCS's potential in reducing neurological complications during ECMO support in high-risk patients.

Neurological complications significantly worsen outcomes in cardiogenic shock (CS) patients on ECMO. Previous studies have reported that VA-ECMO carries a higher neurological risk than venovenous ECMO (8, 18, 19). More importantly, patients with ECMO-related adverse neurological events may have a two- to three-fold increase in in-hospital mortality (20). In our case, the patient developed a right frontal lobe hemorrhage following cardiac arrest and required VA-ECMO, placing him at high risk for secondary brain injury. The stability of cerebral hemodynamics observed during TCCS-guided flow titration was in alignment with the absence of further neurological deterioration, highlighting the potential utility of real-time neuromonitoring in high-risk ECMO patients. The concomitant use of IABP with VA-ECMO has been shown to reduce left ventricular afterload and improve pulsatile cerebral perfusion compared with VA-ECMO alone, potentially mitigating the risk of secondary brain injury in patients with Postcardiotomy Shock (21). However, this combined approach also introduces additional complexity in hemodynamic interpretation, as both devices independently modulate arterial waveform morphology and cerebral flow indices (22).

TCCS monitoring has shown value across age groups in ECMO patients. In children, altered cerebral flow velocities and elevated non-invasive intracranial pressure (nICP) correlate with outcomes, suggesting nICP as a prognostic marker (23–25). In adults on VA-ECMO, pulsatility index (PI) closely reflects cerebral perfusion status: low PI often indicates impaired pulsatility due to severe left ventricular dysfunction, while rising PI may signal improving cardiac function or evolving ischemia (26–28). In another previous study, Caturegli et al. found that absent pulsatility on transcranial Doppler was significantly associated with intraparenchymal hemorrhage in VA-ECMO patients, underscoring the potential of Doppler-derived indices as markers of cerebrovascular injury (14). In the present case, the clinical utility of TCCS was directly demonstrated during the critical weaning phase: as ECMO flow was dynamically titrated down from 3.25 L/min to 2.12 L/min, serial TCCS measurements confirmed that PI remained stable within the target range of 0.65–1.1 and nICP stayed between 15.59 and 17.60 mmHg. This real-time feedback allowed clinicians to adjust ECMO flow while maintaining cerebral hemodynamic parameters within target ranges, which may have contributed to the avoidance of secondary ischemic or hemorrhagic events. These findings suggest TCCS is a feasible tool that may support individualized hemodynamic management in adult CS patients on VA-ECMO.

It should be noted that the Czosnyka formula used in this study was originally derived and validated in a cohort of patients with hydrocephalus (17), and its physiological assumptions may not hold under conditions of non-pulsatile or mechanically assisted circulation such as VA-ECMO with IABP support. Moreover, PI and nICP are influenced by assisted circulation, impaired cerebral autoregulation, and fluctuations in MAP, PaCO₂, and CO. These factors, which are inherent to ECMO/IABP physiology, violate key assumptions underlying the original model. Therefore, while nICP provided a useful trend-based reference for flow titration in this case, it should be interpreted as an indirect and assumption-dependent surrogate that may be unreliable under conditions of severely altered pulsatility or circulatory assistance. Other non-invasive ICP monitoring techniques, such as optic nerve sheath diameter (ONSD) ultrasonography, have also shown strong correlation with invasive ICP measurements in traumatic brain injury and may offer complementary information, though their validation in the specific ECMO population remains limited (29). Prospective validation of this or any PI-based nICP model in the specific context of VA-ECMO is essential before clinical generalization.

TCCS, as an advanced monitoring technique, allows visualization of intracranial 2D structures, blood flow, and Doppler spectra in ECMO patients via the temporal window. This provides a more comprehensive assessment of ICP, cerebral hemodynamic status, and regulatory capacity. However, the widespread adoption of TCCS faces challenges, as it is currently mastered by only a few ECMO centers, limiting its broad application in cerebral monitoring for ECMO patients (30). In the present case, the patient received TCCS monitoring throughout the treatment process, and blood flow was adjusted according to TCCS-derived parameters. The results showed that TCCS could dynamically provide blood flow perfusion information and should be tested in future large-scale population studies.

Current evidence suggests that while neuromonitoring cannot directly prevent acute brain injury from occurring, its clinical significance during ECMO support is substantial (15). However, continuous and effective neuromonitoring facilitates the early detection of abnormalities in cerebral hemodynamics and oxygenation, providing a basis for timely clinical intervention and potentially improving neurological outcomes at discharge (16).

Future research should focus on utilizing TCCS to guide the management of intracranial blood flow in patients with impaired autoregulation. There is a need to deepen the understanding of cerebral hemodynamic changes under ECMO support, conduct high-quality clinical trials to validate the efficacy and reliability of neuromonitoring techniques, define their role in clinical decision-making, and establish standardized and effective cerebral protection strategies to further enhance patient survival and quality of life.

However, the case report nature limited the generalizability of the findings. The observed benefits of TCCS-guided management may be influenced by institutional expertise and protocol-specific factors. Future validation through larger, multicenter prospective studies with diverse patient populations is essential to establish standardized protocols and confirm the neuroprotective impact of TCCS in ECMO-supported cardiogenic shock.

## Conclusion

This case showed that TCCS might enable real-time, non-invasive monitoring of cerebral hemodynamics in VA-ECMO–supported cardiogenic shock patients. TCCS enabled real-time, non-invasive monitoring of cerebral hemodynamics in a patient with VA-ECMO–supported cardiogenic shock. It provided actionable data that informed ECMO flow titration to maintain target cerebral perfusion parameters. Wider adoption and validation through multicenter studies are needed to establish its role in neuroprotective strategies.

## Data Availability

The original contributions presented in the study are included in the article/Supplementary Material, further inquiries can be directed to the corresponding author.
