# Supplementary material for: A case report of cardiogenic shock with transcranial color-coded ultrasound monitoring under ECMO
Source: Front Cardiovasc Med. 2026 Sep 3;13:1844563. doi: 10.3389/fcvm.2026.1844563 (PMC13581902; doi:10.3389/fcvm.2026.1844563)
Supplement: Supplementary file 1 [file Datasheet1.docx]

**Table S1. Serial Hemodynamic Parameters, Vasoactive Drug Doses, and ECMO Settings**

| **Date** | **Time** | **SBP**  **(mmHg)** | **DBP**  **(mmHg)** | **PaCO2/PaO2** | **Hb**  **(g/L)** | **Ventilator settings** | **Dopamine**  **(mL)** | **Norepinephrine**  **(mL)** | **ECMO Speed**  **(rpm)** | **ECMO Flow**  **(L/min)** |
| --- | --- | --- | --- | --- | --- | --- | --- | --- | --- | --- |
| 15-Aug | 11:00 | 60 | 30 | 42/483 | 109 | A/C | 8 | 8 | 3220 | 3.33 |
|  | 12:00 | 60 | 40 | — | — | A/C | 8 | 8 | 3220 | 3.07 |
|  | 13:00 | 74 | 40 | — | — | A/C | 8 | 8 | 3225 | 3.01 |
|  | 14:00 | 71 | 39 | — | — | A/C | 8 | 8 | 3225 | 3.27 |
|  | 15:00 | 73 | 40 | — | — | A/C | 8 | 8 | 3225 | 3.2 |
|  | 16:00 | 79 | 47 | 41/114 | — | A/C | 8 | 8 | 3225 | 3.15 |
|  | 17:00 | 82 | 50 | 38/172 | — | A/C | 8 | 8 | 3225 | 3.09 |
|  | 18:00 | 86 | 56 | — | — | A/C | 8 | 8 | / | / |
|  | 19:00 | 90 | 70 | — | — | A/C | 8 | 8 | / | / |
|  | 20:00 | 102 | 76 | — | — | A/C | 8 | 5 | 3090 | 2.75 |
|  | 21:00 | 108 | 80 | — | — | A/C | 8 | 5 | 3075 | 2.92 |
|  | 22:00 | 101 | 78 | — | — | A/C | 8 | 5 | 3075 | 2.96 |
|  | 23:00 | 118 | 79 | — | — | A/C | 8 | 5 | 3075 | 2.95 |
| 16-Aug | 0:00 | 121 | 81 | 38/241 | 83 | A/C | 8 | 5 | 2885 | 2.45 |
|  | 1:00 | 119 | 82 | — | — | A/C | 8 | 5 | 2885 | 2.49 |
|  | 2:00 | 127 | 85 | — | — | A/C | 8 | 5 | 2885 | 2.55 |
|  | 3:00 | 115 | 79 | — | — | A/C | 8 | 5 | 2885 | 2.37 |
|  | 4:00 | 111 | 81 | — | — | A/C | 8 | 5 | 2885 | 3.34 |
|  | 5:00 | 109 | 82 | — | — | A/C | 8 | 5 | 2885 | 2.57 |
|  | 6:00 | 111 | 80 | — | — | A/C | 8 | 5 | 2885 | 2.52 |
|  | 7:00 | 112 | 79 | 38/172 | — | A/C | 8 | 5 | 2885 | 2.54 |
|  | 8:00 | 88 | 52 | — | — | A/C | 5 | 2.5 | 3110 | 3.01 |
|  | 9:00 | 88 | 50 | — | — | A/C | 5 | 1 | 3110 | 3.08 |
|  | 10:00 | 114 | 50 |  | 89 | A/C | 5 | 2 | 3190 | 3.1 |
|  | 11:00 | 107 | 62 | — | — | A/C | 5 | 3 | 3190 | 3.08 |
|  | 12:00 | 124 | 58 | — | — | A/C | 5 | 2 | 3185 | 3.05 |
|  | 13:00 | 116 | 53 | — | — | A/C | 5 | 2 | 3185 | 3.04 |
|  | 14:00 | 130 | 55 | 56/289 |  | A/C | 5 | 2 | 3185 | 3.17 |
|  | 15:00 | 109 | 54 |  | 78 | A/C | 5 | 1 | 3185 | 2.98 |
|  | 16:00 | 110 | 56 | — | — | A/C | 5 | 1 | 3185 | 3.01 |
|  | 17:00 | 103 | 52 | 45/247 | — | A/C | 5 | 1 | 3185 | 3.15 |
|  | 18:00 | 100 | 55 | 50/233 | — | A/C | 5 | 1 | 3185 | 3.24 |
|  | 19:00 | 107 | 51 | — | 85 | A/C | 5 | 1 | 3185 | 3.16 |
|  | 20:00 | 111 | 68 | — | — | A/C | 5 | 1 | 3185 | 3.17 |
|  | 21:00 | 110 | 58 | — | — | A/C | 5 | 1 | 3185 | 3.18 |
|  | 22:00 | 114 | 50 | — | — | A/C | 5 | 1 | 3185 | 3.08 |
|  | 23:00 | 109 | 54 | 35/345 | 87 | A/C | 5 | 1 | 3185 | 3.1 |
| 17-Aug | 8:00 | 110 | 40 | 36/283 | — | SIMV | 5 | 0.6 | 3218 | 3.12 |
|  | 9:00 | 122 | 48 |  | 91 | SIMV | 5 | 0.6 | 3218 | 3.02 |
|  | 10:00 | 126 | 50 | — | — | SIMV | 5 | 0.6 | 3218 | 3.11 |
|  | 11:00 | 128 | 58 | — | — | SIMV | 5 | 0.6 | 3218 | 3 |
|  | 12:00 | 134 | 70 | — | — | SIMV | 5 | 0.6 | 3218 | 2.98 |
|  | 13:00 | 133 | 72 | — | — | SIMV | 5 | 0.6 | 3218 | 3.2 |
|  | 14:00 | 144 | 79 | 41/174 | 87 | SIMV | 5 | 0.6 | 2885 | 2.12 |
|  | 15:00 | 132 | 76 | — | — | SIMV | 5 | 0.4 | 2885 | 2.04 |
|  | 16:00 | 122 | 66 | — | 86 | SIMV | 5 | 0.4 | / | / |

Note: Dopamine infusion consisted of 180 mg dopamine diluted in 32 mL normal saline.

Norepinephrine infusion consisted of 18 mg norepinephrine diluted in 5% glucose solution (41 mL).

SBP, systolic blood pressure; DBP, diastolic blood pressure; ECMO, extracorporeal membrane oxygenation.

/, missing or unavailable data.

**Table S2. Neurocritical timeline information.**

| Intervention & Support | Date | Time | Examination & TCCS Parameters | Pupils | Brainstem reflexes | Continuous analgesia and sedation | Neurological Status | Anticoagulation strategy |
| --- | --- | --- | --- | --- | --- | --- | --- | --- |
| CPR, AED, endotracheal intubation, epinephrine | 15-Aug | 09:00 | — | Absent | Absent | NONE | GCS 3, coma | no anticoagulation |
| Advanced life support |  | 10:20 | — | Absent | Absent | NONE | Unconscious | no anticoagulation |
| ECMO started; transferred to hospital |  | 10:45 | — | Absent | Absent | NONE | GCS 3 | no anticoagulation |
| CRRT initiated for acidosis |  | 12:00 | Head CT: right frontal lobe hemorrhage | Absent | Absent | YES | GCS 3 | individualized restrictive anticoagulation strategy,unfractionated heparin,APTT <50 seconds |
| Electrical cardioversion |  | 13:00 | — | Absent | Absent | YES | — | individualized restrictive anticoagulation strategy,unfractionated heparin,APTT <50 seconds |
| Balloon angioplasty + stenting |  | 17:00 | CAG: RCA recanalized | constriction recovered | returned | Yes | — | unfractionated heparin,1000u |
| ECMO + IABP + CRRT; vasopressors | 16-Aug | 16:00 | TCCS monitoring started (MCA flow, PI, RI, nICP) | Normal | Normal | Yes | Comatose | no anticoagulation |
| CRRT discontinued | 16-Aug | 19:00 | BP 124/70 mmHg | Normal | Normal | Yes | — | no anticoagulation |
| ECMO weaning per TCCS targets | 17-Aug | 14:00 | TCCS: PI 0.65–1.1; nICP 15.59–17.60 mmHg | Normal | Normal | Yes | Stable | no anticoagulation |
| IABP continued |  | 17:00 | Cerebral perfusion stable | Normal | Normal | Yes | Improved | no anticoagulation |
| No support |  | 17:00 | nICP slightly higher under IABP alone | Normal | Normal | NONE | Consciousness improved | no anticoagulation |
|  |  |  |  |  |  |  |  |  |
| — | 2-Sep | 10:00 | Head CT: hemorrhage partially resolved | Normal | Normal | NONE | Neurological recovery |  |
| — |  |  | Head CT: hemorrhage nearly resolved | Normal | Normal | NONE | — |  |
| Rehabilitation |  |  | — | Normal | Normal | NONE | Independent living |  |
| — |  |  | Hemorrhage fully resolved | Normal | Normal | NONE | Favorable neurological recovery |  |
